# High Agreement Across Laboratories Between Different Alpha‐Synuclein Seed Amplification Protocols

**DOI:** 10.1111/ene.70165

**Published:** 2025-04-16

**Authors:** Stefan Bräuer, Maximilian Weber, Christian Deuschle, Kühlwein Julia, Luis Concha‐Marambio, Alexander M. Bernhardt, Vaibhavi Kadam, David Mengel, Wolfgang P. Ruf, Jan Kassubek, Iñaki Schniewind, Sandra Kuhs, Marcello Rossi, Piero Parchi, Johannes Levin, Karin M. Danzer, Matthis Synofzik, Kathrin Brockmann, Björn H. Falkenburger

**Affiliations:** ^1^ German Center for Neurodegenerative Diseases (DZNE) Dresden Germany; ^2^ Department of Neurology Technische Universität Dresden Germany; ^3^ Department of Neurodegeneration and Hertie‐Institute for Clinical Brain Research University of Tübingen Tübingen Germany; ^4^ German Center for Neurodegenerative Diseases Ulm Germany; ^5^ Department of Neurology Ulm University Ulm Germany; ^6^ Amprion San Diego California USA; ^7^ Department of Neurology Ludwig‐Maximilians‐Universität München Munich Germany; ^8^ German Center for Neurodegenerative Diseases Tübingen Germany; ^9^ German Center for Neurodegenerative Diseases (DZNE) Bonn Germany; ^10^ IRCCS Istituto Delle Scienze Neurologiche di Bologna (ISNB) Bologna Italy; ^11^ Department of Biomedical and Neuromotor Sciences University of Bologna Bologna Italy; ^12^ German Center for Neurodegenerative Diseases (DZNE) Munich Germany; ^13^ Munich Cluster of Systems Neurology (SyNergy) Munich Germany

**Keywords:** alpha‐synuclein, method validation, proficiency testing, ring trial, RT‐QuIC

## Abstract

**Background:**

Seed amplification assays (SAA) detect alpha‐synuclein (aSYN) pathology in patient biomatrices such as cerebrospinal fluid (CSF)—potentially even before clinical manifestations. As CSF‐based SAA are approaching broader use in clinical trials and research, ensuring that different laboratories obtain the same results becomes increasingly important.

**Methods:**

In this cross‐laboratory, cross‐aSYN‐recombinant substrate and cross‐protocol round‐robin test, we compared SAA results from a common set of 38 CSF samples measured independently in four research laboratories of the German Center for Neurodegenerative diseases. Three laboratories (A–C) used an assay protocol adapted from Parchi's group at ISNB (Bologna, Italy); laboratory D used an assay protocol adapted from Amprion Inc. Two different manufacturers of aSYN protein were used as substrates for the SAA reaction.

**Results:**

Qualitative results were identical in at least three of the four laboratories for 37 out of 38 samples (20 positive, 17 negative). Fleiss Kappa for all four laboratories was 0.751 (*z* = 12, *p* < 0.001). For each laboratory, agreement with laboratory A was > 92%. For the number of positive replicates, Fleiss Kappa was 0.45 for a score of zero positive replicates and 0.42 for a score of four positive replicates.

**Conclusions:**

The qualitative SAA results showed a high level of agreement across research laboratories, aSYN monomers, and assay protocols. Small differences between laboratories were systematic, consistent with the notion that SAA reports biologically relevant properties. These results also underline that round‐robin tests can be helpful in assessing and ensuring SAA quality across laboratories.

## 1 Introduction

The deposition of pathologically aggregated forms of alpha‐synuclein (aSYN) as intracellular inclusions is a central component in the pathogenesis of Parkinson's disease (PD) and other neurodegenerative disorders, collectively called synucleinopathies. Seed amplification assays (SAA) have been developed to detect minute amounts of “seeding competent” species of aSYN in cerebrospinal fluid (CSF), brain extracts, and other biomatrices [1, 2, 3, 4] of patients with synucleinopathies. SAA robustly separates patients with PD and dementia with Lewy bodies (DLB) from healthy controls with high sensitivity and specificity [5, 6, 7], and accurately differentiates between PD and multiple system atrophy (MSA) [3, 4, 8, 9].

Fluid biomarkers are increasingly important in clinical research, in particular those that are disease‐specific or identify relevant co‐pathologies. SAA for aSYN may be useful in multiple contexts: patient stratification in clinical trials, potentially even as inclusion or exclusion criteria; measurement of target engagement of pathological aSYN lowering therapies; detection of neurodegenerative diseases in presymptomatic stages; or diagnostic confirmation in patients with Parkinsonian or dementia syndromes of uncertain diagnosis in clinical settings. Consequently, SAA are presently being established in many research and clinical laboratories. Yet, all these applications require SAA results to be reproducible across research laboratories.

One previous study compared different SAA protocols between one academic laboratory and two companies (7). We previously compared quantitative and qualitative SAA results with another established laboratory [10]. However, larger comparative trials of SAA performance across various laboratories—ideally even using different SAA protocols and recombinant aSYN monomers—have been missing.

In the present study, we conducted an aSYN SAA round‐robin test among four laboratories of the German Center for Neurodegenerative Diseases (DZNE). Three laboratories used an assay protocol adapted from Parchi's group at ISNB [3]. This assay is positive for neuronal aSYN diseases such as PD and DLB and negative for MSA. One laboratory used an assay protocol adapted from Amprion Inc. [9], which is positive for PD, DLB, and MSA, with quantitative differences in the kinetics of the maximum fluorescence intensity between PD/DLB and MSA. Two different aSYN monomers were used. CSF of 38 study participants was included, aliquots of the same samples were sent to each laboratory in a blinded fashion, and unblinding was performed after all measurements were completed. Our design thus allowed inferences about the implementation of the same protocol in different laboratories, including the same aSYN monomer, and about differences between SAA protocols also using different aSYN monomers.

## 2 Methods and Materials

The SAA were carried out independently and blinded to clinical diagnosis in laboratories of RG Falkenburger at DZNE Dresden (laboratory A), RG Brockmann at DZNE Tübingen (laboratory B), RG Danzer at DZNE Ulm (laboratory C), and RG Synofzik at DZNE Tübingen (laboratory D).

### 2.1 Study Population

CSF was collected from consenting study participants with Parkinson's disease (PD, *n* = 14), Dementia with Lewy bodies (DLB, *n* = 4) or controls (Ctrl., *n* = 20) who underwent lumbar puncture as part of routine diagnostics at each study site or at DZNE Munich. Demographic information is summarized in Table 1. The lumbar punctures were performed according to standard protocols. CSF samples were centrifuged and frozen at −80°C within 90 min of collection. The diagnosis of PD was made according to the Movement Disorders Society Criteria [11]; DLB was diagnosed according to the DLB consortium revised consensus criteria [12]. Control CSF samples were selected from patients without clinical signs of aSYN pathology. The study was approved by the local ethics committees (Dresden: BO‐EK‐444092021, Tuebingen:199/2011BO1 and 353/2022BO2; Ulm: 20/10 and 405/19; Munich 23–0602).

**TABLE 1:** Patient demographics.

	Positive controls^a^	Negative controls^b^
Sex (female/male)	7/11	8/12
Age at lumbar puncture in years	65.4 (13.2)	64.6 (14.5)
Disease duration in years	5.4 (4.8)	n.a.
Hoehn and Yahr stage	2.5 (1.2)	n.a.
UPDRS part 3	28.4 (7.8)	n.a.

^a^ 14 patients were diagnosed with Parkinson's disease, four patients with dementia with Lewy bodies.

^b^ Patients with diagnoses such as tension headache, functional disorders, transient ischaemic attack, and idiopathic intracranial hypertension. Data depicted as mean (standard deviation).

### 2.2 Blinding

From each DZNE site, 8–12 CSF samples (4–6 from study participants with Lewy body diseases and an equal number of controls) were sent to DZNE Bonn, where they were aliquoted and re‐labeled for blinding. The entire set of 38 aliquots was sent to each laboratory for blinded analysis. Results were collected at DZNE Bonn. Unblinding and data analysis was performed after all results had been obtained.

### 2.3 Human α‐Synuclein Monomer

At laboratory A, aSYN monomer was generated and purified as previously described [10, 13]. Briefly, BL21 (DE3) *E. coli* bacteria (Thermo Fischer Scientific) were transformed with the vector plasmid containing the WT human aSYN with N‐terminal His‐tag. Expression was induced by an autoinduction medium, and cells were harvested after 16 h. The cell pellet was lysed via osmotic shock [400 g/L sucrose (Carl Roth), 30 mM TRIS (Carl‐Roth) pH 7.2, 2 mM EDTA (Thermo Fischer Scientific)]. The solution was centrifuged and the pellet resolved in water. After centrifugation, the supernatant's pH was reduced to 3.5. The solution was centrifuged again and the supernatant's pH increased to 7.5. Next, we performed immobilized metal ion affinity chromatography using an NGC chromatography system (BioRad) and HisTrap FF column (Cytivia). The selected fraction was loaded on a HiTrap Q‐HP anion exchange column (Cytivia); the selected fractions were pooled and subsequently dialyzed against water using a 3.5 kDa MWCO dialysis membrane (Thermo Fisher Scientific) overnight at 4°C. The protein concentration was measured using a spectrometer (NanoDrop, Thermo Fischer Scientific). Using this protocol, the average yield from 1 L of bacteria culture was on the order of 55 mg aSYN. The samples were aliquoted in 0.7 mg portions and stored at −80°C until further use.

The aSYN monomer produced at laboratory A was used in laboratories A, B, and C. All three laboratories used the same batch of aSYN (~55 mg). Laboratory D used an aSYN monomer provided by Amprion Inc.

### 2.4 α‐Synuclein Seed Amplification Assays

At laboratories A, B, and C, the SAA was performed as previously described [10]. In brief, measurements were performed using a black 96‐well plate (Thermo Fischer Scientific). Each well contained six 0.8 mm silica beads (OPS Diagnostics). Per well, 15 μL of CSF was added to 85 μL of reaction buffer, which contained 40 mM phosphate buffer (Carl Roth) pH 8.0, 0.0015% sodium dodecyl sulfate (SDS) (Carl Roth), 10 μM Thioflavin T (Carl Roth), 0.1 mg/mL recombinant aSyn, and 170 mM NaCl (Carl Roth). The plate was incubated in a BMG FLUOstar Omega plate reader at 42°C with cycles of 1 min double orbital shaking (400 rpm) and 1 min rest. The fluorescence measurements were performed every 45 min.

Each sample was measured as four technical replicates on the same plate. Each plate included at least two negative and two positive controls, each with four replicates. Relative fluorescence units (RFU) for every time point were expressed as a percentage of the maximum intensity reached on that plate. A replicate was considered positive if fluorescence crossed a threshold within 40 h. The fluorescence threshold was defined as the average intensity of previously measured negative controls during the first 10 h of recording, plus 40 standard deviations.

In laboratories A and B, samples were considered positive if at least two out of four replicates were positive. Samples were considered negative if no replicate out of four was positive. Samples were run again if one replicate out of four was positive. In the phase of initial assay implementation, prior to this study, laboratory C had a higher rate of false positive replicates analyzing a subset of samples not part of this study. They therefore decided to adjust the analysis protocol for this round robin trial. Therefore, samples in laboratory C were considered positive if at least three out of four replicates were positive, or resulted in two out of four positive replicates in three consecutive assay runs. Samples were considered negative if no or one replicate out of four was positive. Samples were run again if two replicates out of four were positive for up to three times. One sample showed two out of four positive replicates in two assay runs, but could not be measured a third time because the CSF amount was too low. This sample was defined as inconclusive.

At laboratory D, the SAA was carried out as described before [9]. In brief, two 1/8″ silicon nitride (Si3N4) beads (grade 5) (Tsubaki Nakashima) were added per well with an Amprion‐designed bead dispenser. 40 μL of CSF samples were loaded in three technical replicates in clear flat bottom black 96‐well microplates (#655906, Greiner), to which 60 μL of substrate reaction mix consisting of 0.3 mg/mL recombinant monomeric human aSYN substrate (#S2020, Amprion), 10 μM ThT (#T3516, Millipore‐Sigma), 0.1% Sarkosyl (#61747‐100ML, Millipore‐Sigma), 100 mM PIPES (#80635, Millipore Sigma) pH 6.5 adjusted using 1 M NaOH, 0.44 M NaCl (#1064040500, Sigma Aldrich) was added. Samples were measured using the FLUOstar Omega plate reader (BMG Labtech) with an incubator set at 42°C, using excitation and emission filters at 440–10 and 490–10 nm. The fluorescence of this instrument was calibrated using the Atto425 dye, allowing absolute fluorescence intensities to be analyzed. The plate was shaken for 1 min at 800 rpm (orbital motion) every 15 min before each of the 97 total cycles. After run completion, data was analyzed using MARS Omega data analysis software 4.00 R2 (BMG Labtech), and the qualitative and synucleinopathy results were reported based on the *F*_max_ reading (highest fluorescence recorded per well) of all triplicates of a sample. Thus, a replicate with *F*_max_ less than the positive threshold (3000 RFU) was considered negative, one with *F*_max_ equal to or higher than the positive threshold (3000 RFU) but lower than the LBD threshold (45,000 RFU) was considered Type 2, and with F_max_ equal to or higher than the LBD threshold was considered Type 1. If all three or two out of three replicates were negative, then the samples were reported negative. If all three or two out of three replicates were Type 2, then the samples were reported as positive (qualitative status) and Type 2 (seed status). If all three replicates were Type 1, then the sample was reported as positive with Type 1 seeds. If two out of three replicates were Type 1 and the third was Type 2, then the sample was reported as positive and undetermined. If two out of three replicates were Type 1, and the third was negative or if all three replicates were different, then the samples were reported as inconclusive/undetermined.

Representative Traces are depicted in Figure S1.

### 2.5 Statistical Analysis

Data analysis was conducted using Rstudio (version 2023.06.2 + 561). R code can be obtained from the corresponding author upon request. Fleiss Kappa was calculated using the “kappam.fleiss” function of the “irr” library. Shapiro‐Wilk test was used to test for normal distribution. Pearson correlation was used for normally distributed data. Spearman's rho was used for correlation of non‐normally distributed data. The entire dataset is included as Table S1.

## 3 Results and Discussion

### 3.1 Agreement of Qualitative Findings

**FIGURE 1:**
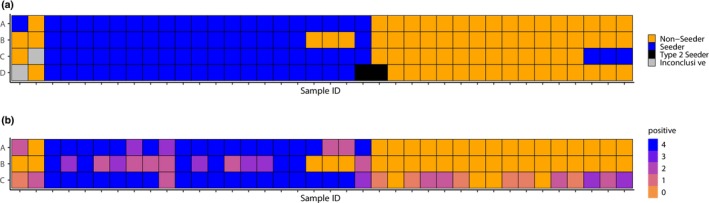
(a) Qualitative results from SAA measurements of 38 samples in 4 different laboratories. Results from laboratories A–C were either “Non‐Seeder” or “Seeder”; results from laboratory D were “Non‐Seeder” or “(Type 1) Seeder” or “Type 2 Seeder”. Two measurements of distinct samples were inconclusive by one laboratory (gray). (b) Quantitative results. In laboratories A–C, each sample was run as quadruplicates. The number of positive replicates for each sample is color‐coded.

The qualitative findings were highly consistent between laboratories; results were identical in at least three of the four laboratories for 37 out of 38 samples and identical in all four laboratories for 28 out of 38 samples (Figure 1a). Accordingly, the Fleiss Kappa for qualitative results from all four laboratories was 0.75 (substantial agreement, *z* = 11.8, *p* value < 0.001).

### 3.2 Agreement of Quantitative Findings

The three laboratories (A–C) that used the same SAA protocol and aSYN monomer measured the samples as quadruplicates [3]. We, therefore, compared the number of positive replicates as an additional, quantitative readout (Figure 1b). The overall Fleiss Kappa for the quantitative findings was 0.28 (fair agreement), but it was 0.45 for a score of zero positive replicates and 0.42 for a score of four positive replicates, indicating that the results of 0 and 4 positive replicates were measured more reliably across laboratories than the intermediate results of 2 and 3 positive replicates.

We noted that the differences between laboratories A–C were not random, but systematic. Laboratory B obtained an intermediate number of positive replicates for several samples for which laboratories A and C obtained four positive replicates (Figure 1b). Conversely, laboratory C obtained an intermediate number of positive replicates for several samples for which laboratories A and B obtained zero positive replicates (Figure 1b). Accordingly, laboratory B obtained “non‐seeder” results for three samples for which all other laboratories obtained “seeder” results (Figure 1a); laboratory C obtained “seeder” results for three samples for which all other laboratories obtained “non‐seeder” results (Figure 1a). Both laboratories did not show deviations in the other direction, that is, laboratory B never obtained “seeder” results when other laboratories did not, and laboratory C never obtained “non‐seeder” results when other laboratories did not. When round‐robin trials are implemented to ensure that SAA findings are comparable across laboratories, the threshold to detect seeding in laboratory B could be slightly lowered, and the threshold in laboratory C could be slightly increased.

These systematic differences between laboratories suggest that the amplification efficiency may be affected by minor modifications in the way the assay is performed, such as the source of buffer reagents or the individual steps of handling. Between samples, the property to be “even detected with somewhat less efficient amplification” or “only detected with the most efficient amplification” are likely biological properties of the samples and not random effects. For instance, seeding could be more difficult to detect in samples with fewer seeds, and the sensitivity of the assay might differ slightly between laboratories. These findings are consistent with the accumulating evidence that SAA can detect biologically relevant quantitative differences between patients. For instance, we recently observed a correlation between kinetic parameters and cognitive performance cross‐sectionally [10] and longitudinally [14]. Moreover, SAA fluorescence intensity showed a response to aSYN antibody treatment in patients [15].

### 3.3 Agreement of Kinetic Parameters

**FIGURE 2:**
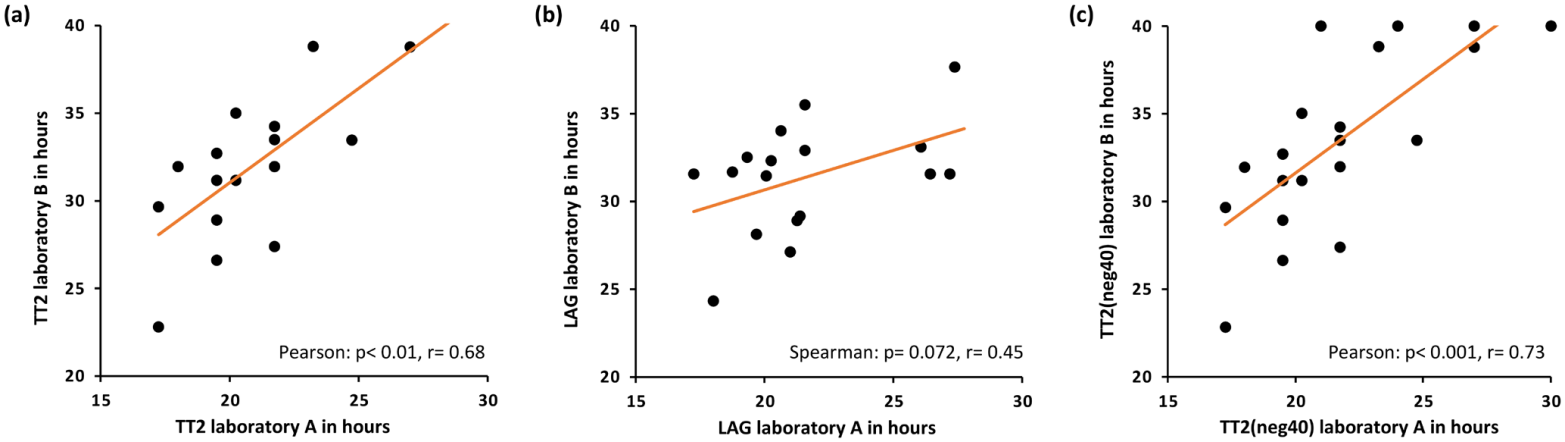
Correlation of kinetic parameters between laboratories. (a) Correlation of the second fastest lag phase (TT2) measured in laboratory A with TT2 measured in laboratory B. (b) Correlation of average lag phase (LAG) measured in laboratory A with LAG measured in laboratory B. (c) For samples positive in one laboratory and negative in the other, TT2 was set to 40 h (TT2neg40). These modified values for TT2 were then correlated between laboratories A and B. In panels (a–c), each marker represents one patient sample.

For two laboratories (A and B), kinetic assay parameters were available, namely average Lag‐phase (LAG) and “Time to threshold 2” (TT2). TT2 describes the second shortest Lag‐phase of a sample and represents a median‐like feature. In a previous study we observed that the TT2 better represented patient heterogeneity than LAG [10]. We found a moderate positive correlation when comparing the TT2 between laboratory A and B (Pearson *p* < 0.01, *r* = 0.68) (Figure 2a). The comparison of the LAG (Figure 2b) showed only a tendency for a weak positive correlation (Spearman *p* = 0.072, *r* = 0.45). This indicates that kinetic parameters can be comparable between laboratories, at least when the same SAA protocol and the same aSYN batch are used. Especially the TT2 could be a robust parameter that enables the comparison of kinetic results.

We suggest that the comparability of kinetic assay parameters could be further improved by imputing the TT2 for negative samples by setting it to the maximum value of 40 h, resulting in a strong positive correlation (Pearson *p* < 0.001, *r* = 0.73) (Figure 2c). This will allow the inclusion of such samples in inter‐laboratory comparisons and the identification of systematic differences. Furthermore, including reference samples for kinetic parameters could be used to compensate for variability between individual plates.

### 3.4 Agreement for Different Diagnoses

Finally, we compared SAA findings within the three diagnostic groups of CSF samples, PD, DLB, and Ctrl. Results were most consistent for DLB, with “seeder” results for all four samples in all four laboratories (Figure S2a), suggesting a sensitivity for DLB close to 100%, in line with previous findings [1, 3, 4, 7, 16].

For the 14 samples with a clinical diagnosis of PD, 11 showed “seeder” results in all four laboratories and 13 showed “seeder” results in 3 or more laboratories. Samples from one patient with PD showed highly inconsistent results, suggesting that the patient might have atypical PD. With this sample excluded, 50 out of the 52 measurements with samples from patients with PD showed “seeder” results, suggesting sensitivity for PD of 96%, similar to previous findings [1, 3, 4, 7, 16].

For the 20 controls, 3 showed “seeder” results in 3 or 4 laboratories, suggesting that about 15% of patients with a neurological condition that warrants lumbar puncture show evidence of Lewy pathology in CSF. Overall, 65 measurements from controls showed “non‐seeder” results, 13 “seeder”, 1 “Type 2 seeder”, 1 “inconclusive”. Specificity was estimated on the order of 83%, which is in the range previously reported [1, 3, 4, 7, 16].

## 4 Conclusions

Overall, this study documents a high rate of agreement of qualitative SAA findings across laboratories, monomers, and protocols. Quality control measures will be required for future clinical applications. These measures could include round‐robin tests and sending inconclusive samples to reference laboratories. Minor discrepancies between the participating laboratories in this study likely resulted from sample properties and not random effects. These discrepancies are, therefore, not expected to significantly hinder scientific investigation of seeding properties in different patient populations. Adjusted assays and normalization strategies are needed to compare kinetic parameters between laboratories.

## Author Contributions

**Stefan Bräuer:** conceptualization, investigation, writing – original draft, methodology, visualization, writing – review and editing, formal analysis, data curation. **Maximilian Weber:** investigation, writing – review and editing. **Christian Deuschle:** investigation, writing – review and editing. **Kühlwein Julia:** resources, writing – review and editing. **Luis Concha‐Marambio:** methodology, writing – review and editing. **Alexander M. Bernhardt:** resources, investigation. **Vaibhavi Kadam:** investigation, writing – review and editing. **David Mengel:** investigation, writing – review and editing. **Wolfgang P. Ruf:** resources, writing – review and editing. **Jan Kassubek:** resources, writing – review and editing. **Iñaki Schniewind:** investigation, resources, writing – review and editing. **Sandra Kuhs:** resources, investigation, writing – review and editing. **Marcello Rossi:** methodology, writing – review and editing. **Piero Parchi:** methodology, writing – review and editing. **Johannes Levin:** resources, writing – review and editing. **Karin M. Danzer:** resources, investigation, conceptualization, writing – review and editing, supervision. **Matthis Synofzik:** investigation, conceptualization, writing – review and editing, resources, supervision. **Kathrin Brockmann:** conceptualization, investigation, resources, writing – review and editing, supervision. **Björn H. Falkenburger:** conceptualization, investigation, writing – original draft, project administration, data curation, formal analysis, writing – review and editing, funding acquisition, methodology, visualization, supervision, resources.

## Ethics Statement

The study was approved by the local ethic committees (Dresden: BO‐EK‐444092021, Tuebingen: 199/2011BO1 and 353/2022BO2; Ulm: 20/10 and 405/19; Munich 23‐0602).

## Conflicts of Interest

Matthis Synofzik has received consultancy honoraria from Ionis, UCB, Prevail, Orphazyme, Biogen, Servier, Reata, GenOrph, AviadoBio, Biohaven, Zevra, Lilly, and Solaxa, all unrelated to the present manuscript. Luis Concha‐Marambio is an employee of Amprion, a biotechnology company with intellectual property to commercialize SAA technology for diagnostic purposes. He is an inventor of several SAA‐related patents and received grants from the Michael J. Fox Foundation for Parkinson's Research (MJFF‐021233, MJFF‐025017, MJFF‐024735). Kathrin Brockmann received Research Grants from the Michael J Fox Foundation for Parkinson's Research (“LRRK2 Kinase Activity”, “Influence of Inflammatory Profiles on PD Phenotype and Progression”, “Prevent Dementia in GBA‐associated PD”, MJFF PRKN/PINK1 Consortium, MJFF GBA1 Consortium on Joint Analysis, MJFF Grant Biology is the Disease), from the University of Tuebingen (“Endophenotyping of GBA‐PD”), from the German Society for Parkinson DPG, from the Health Forum Baden Wuerttemberg (“Predictive Diagnostic of immune‐associated diseases for personalized medicine”), from the Else Kröner Fresenius Stiftung (“ClinBrain”), and from the German Research Foundation DFG (“CORO‐TREND”). She serves on advisory boards for F. Hoffmann‐La Roche Ltd. and VanqaBio. She received speaker honoraria from Abbvie, Lundbeck, UCB and Zambon. Björn Falkenburger reports speaker honoraria from AbbVie, Stadapharm, Desitin, Zambon, and Bial, all unrelated to the present manuscript. The other authors declare no conflicts of interest.

## Supporting information


Appendix S1.

## Data Availability

The data that support the findings of this study are available from the corresponding author upon reasonable request.
