# Supplementary material for: High Agreement Across Laboratories Between Different Alpha‐Synuclein Seed Amplification Protocols
Source: Eur J Neurol. 2025 Apr 16;32(4):e70165. doi: 10.1111/ene.70165 (PMC12000918; doi:10.1111/ene.70165)
Supplement: Supplementary file 1 — Appendix S1. [file ENE-32-e70165-s001.zip › supplemental table 1.docx]

Supplemental Table 1. Results of Figure 1

| **Clinical**  **Diagnosis** | **Qualitative findings (seeder/non-seeder)** | | | | | **Quantitative findings (positive replicates)** | | |
| --- | --- | --- | --- | --- | --- | --- | --- | --- |
|  | **A** | **B** | **C** | **D** | **Agreement** | **A** | **B** | **C** |
| Ctrl. | Non-Seeder | Non-Seeder | Seeder | Non-Seeder | 3 | 0 | 0 | 2 |
| Ctrl. | Non-Seeder | Non-Seeder | Seeder | Non-Seeder | 3 | 0 | 0 | 3 |
| Ctrl. | Non-Seeder | Non-Seeder | Seeder | Non-Seeder | 3 | 0 | 0 | 3 |
| Ctrl. | Non-Seeder | Non-Seeder | Inconclusive | Non-Seeder | 3 | 0 | 0 | 2 |
| Ctrl. | Non-Seeder | Non-Seeder | Non-Seeder | Type 2 Seeder | 3 | 0 | 0 | 1 |
| Ctrl. | Non-Seeder | Non-Seeder | Non-Seeder | Non-Seeder | 4 | 0 | 0 | 2 |
| Ctrl. | Non-Seeder | Non-Seeder | Non-Seeder | Non-Seeder | 4 | 0 | 0 | 1 |
| Ctrl. | Non-Seeder | Non-Seeder | Non-Seeder | Non-Seeder | 4 | 0 | 0 | 0 |
| Ctrl. | Non-Seeder | Non-Seeder | Non-Seeder | Non-Seeder | 4 | 0 | 0 | 1 |
| Ctrl. | Non-Seeder | Non-Seeder | Non-Seeder | Non-Seeder | 4 | 0 | 0 | 1 |
| Ctrl. | Non-Seeder | Non-Seeder | Non-Seeder | Non-Seeder | 4 | 0 | 0 | 0 |
| Ctrl. | Non-Seeder | Non-Seeder | Non-Seeder | Non-Seeder | 4 | 0 | 0 | 0 |
| Ctrl. | Non-Seeder | Non-Seeder | Non-Seeder | Non-Seeder | 4 | 0 | 0 | 1 |
| Ctrl. | Non-Seeder | Non-Seeder | Non-Seeder | Non-Seeder | 4 | 0 | 0 | 2 |
| Ctrl. | Non-Seeder | Non-Seeder | Non-Seeder | Non-Seeder | 4 | 0 | 0 | 2 |
| Ctrl. | Non-Seeder | Non-Seeder | Non-Seeder | Non-Seeder | 4 | 0 | 0 | 1 |
| Ctrl. | Non-Seeder | Non-Seeder | Non-Seeder | Non-Seeder | 4 | 0 | 0 | 0 |
| Ctrl. | Seeder | Non-Seeder | Seeder | Seeder | 3 | 2 | 0 | 4 |
| Ctrl. | Seeder | Non-Seeder | Seeder | Seeder | 3 | 2 | 0 | 4 |
| Ctrl. | Seeder | Seeder | Seeder | Seeder | 4 | 4 | 4 | 4 |
| DLB | Seeder | Seeder | Seeder | Seeder | 4 | 4 | 3 | 4 |
| DLB | Seeder | Seeder | Seeder | Seeder | 4 | 4 | 2 | 4 |
| DLB | Seeder | Seeder | Seeder | Seeder | 4 | 4 | 4 | 4 |
| DLB | Seeder | Seeder | Seeder | Seeder | 4 | 4 | 3 | 4 |
| PD | Seeder | Non-Seeder | Non-Seeder | Inconclusive | 2 | 2 | 0 | 1 |
| PD | Seeder | Seeder | Seeder | Seeder | 4 | 3 | 2 | 2 |
| PD | Seeder | Seeder | Seeder | Seeder | 4 | 3 | 2 | 4 |
| PD | Seeder | Seeder | Seeder | Type 2 Seeder | 3 | 4 | 2 | 3 |
| PD | Seeder | Non-Seeder | Seeder | Seeder | 3 | 4 | 0 | 4 |
| PD | Seeder | Seeder | Seeder | Seeder | 4 | 4 | 4 | 4 |
| PD | Seeder | Seeder | Seeder | Seeder | 4 | 4 | 3 | 4 |
| PD | Seeder | Seeder | Seeder | Seeder | 4 | 4 | 4 | 4 |
| PD | Seeder | Seeder | Seeder | Seeder | 4 | 4 | 2 | 4 |
| PD | Seeder | Seeder | Seeder | Seeder | 4 | 4 | 3 | 4 |
| PD | Seeder | Seeder | Seeder | Seeder | 4 | 4 | 2 | 4 |
| PD | Seeder | Seeder | Seeder | Seeder | 4 | 4 | 4 | 4 |
| PD | Seeder | Seeder | Seeder | Seeder | 4 | 4 | 3 | 4 |
| PD | Seeder | Seeder | Seeder | Seeder | 4 | 4 | 4 | 4 |
