# Supplementary figures and images for: High Agreement Across Laboratories Between Different Alpha‐Synuclein Seed Amplification Protocols
Source: Eur J Neurol. 2025 Apr 16;32(4):e70165. doi: 10.1111/ene.70165 (PMC12000918; doi:10.1111/ene.70165)

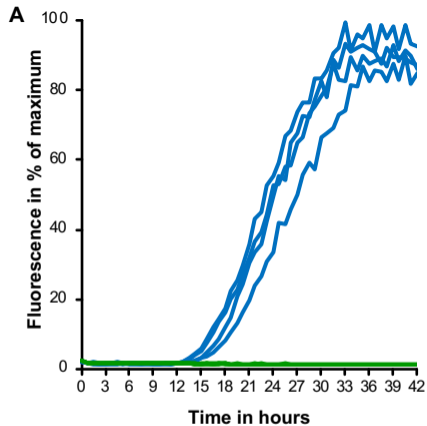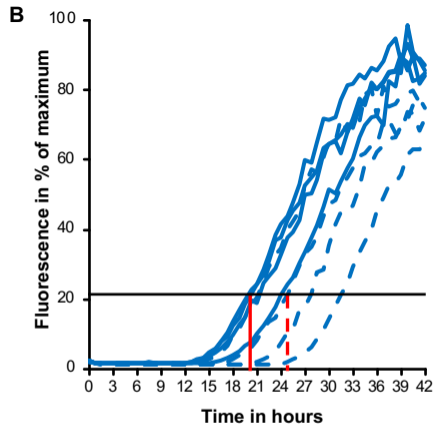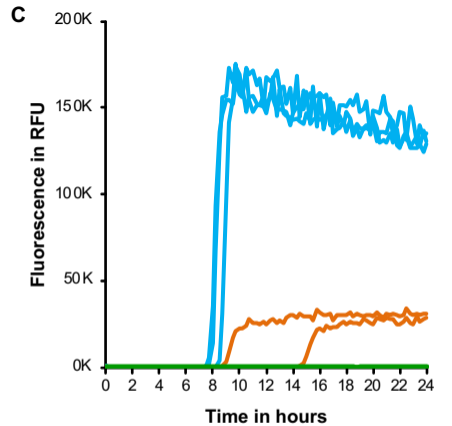

Supplement: Supplementary file 1 — Appendix S1. [file ENE-32-e70165-s001.zip › supplemental figure 1.pdf]

(a)

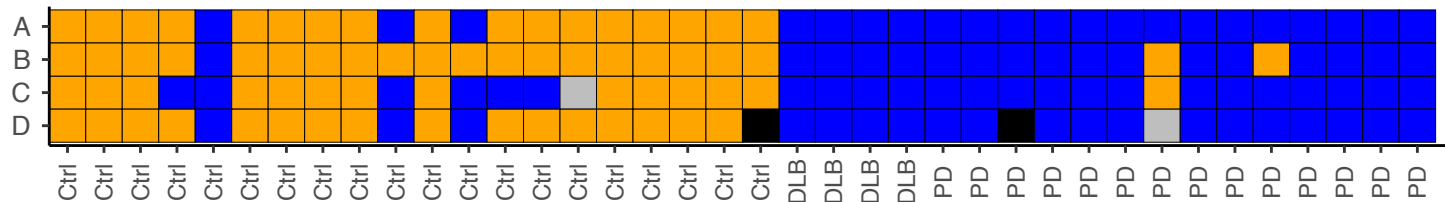

Non-Seeder  
Seeder  
Type 2 Seeder  
Inconclusive

(b)

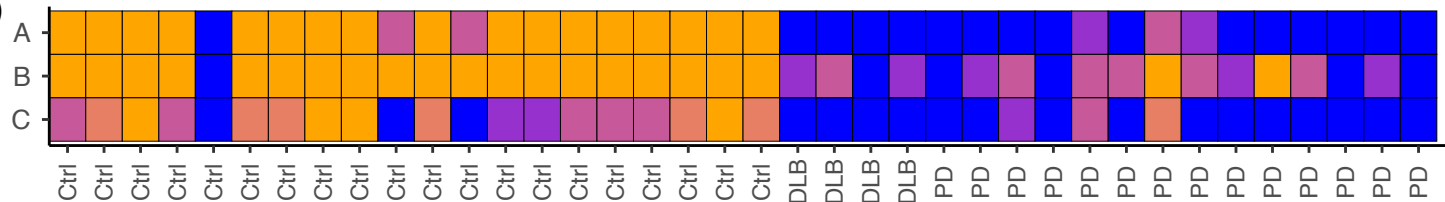

positive

4  
3  
2  
1  
0

Supplement: Supplementary file 1 — Appendix S1. [file ENE-32-e70165-s001.zip › supplemental figure 2.pdf]
